# Supplementary material for: Characterizing the ZrC(111)/c-ZrO2(111) Hetero-Ceramic Interface: First Principles DFT and Atomistic Thermodynamic Modeling
Source: Molecules. 2022 May 5;27(9):2954. doi: 10.3390/molecules27092954 (PMC9100914; doi:10.3390/molecules27092954)
Supplement: Supplementary file 1 [file molecules-27-02954-s001.zip › molecules-1671547-supplementary.pdf]

Supporting Information for

Characterizing the ZrC(111)/*c*-ZrO_2(111) Hetero-ceramic Interface: First Principles DFT and Atomistic Thermodynamic Modeling

Eric Osei-Agyemang,[1] Jean-François Paul,[2] Romain Lucas,[3*] Sylvie Foucaud,[3] Sylvain Cristol[2], Anne-Sophie Mamede[2], Nicolas Nuns[2], Ahmed Addad[4]

[1] Department of Materials Design and Innovation, University at Buffalo, Buffalo, New York 14260-1660, USA

[2] Université de Lille 1, Univ. Lille, CNRS, ENSCL, Centrale Lille, Univ. Artois, UMR 8181 - UCCS - Unité de Catalyse et de Chimie du Solide.

[3] Université de Limoges, IRCER, UMR 7315, F-87068 Limoges, France

[4] Unité Matériaux et Transformations, Université Lille 1, CNRS-UMR 8207

TEM-ED characterization of ZrC/ZrO_2

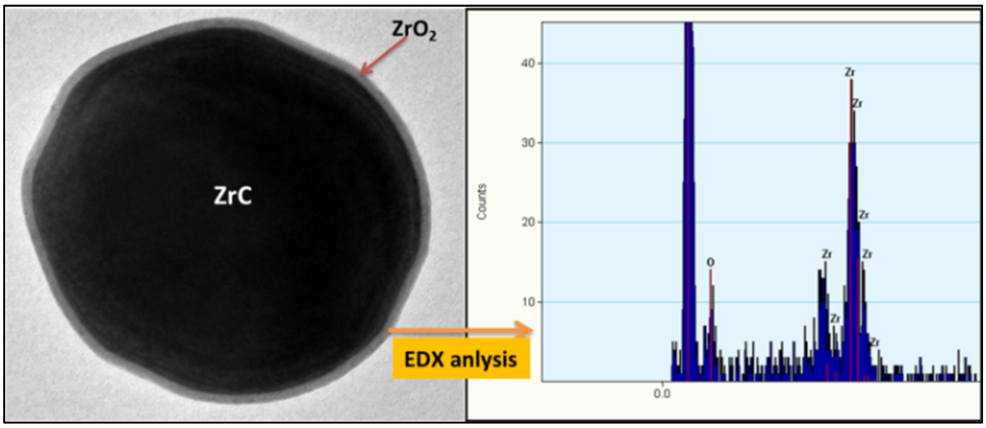

Figure S1. TEM and EDX identification of Zirconium Oxide on ZrC particles.

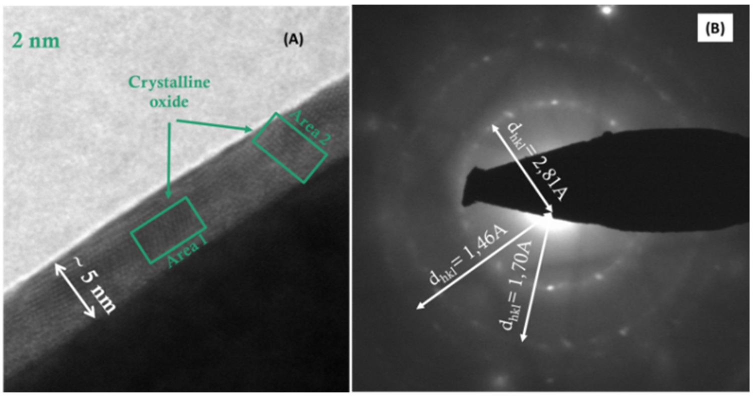

Figure S2. ED pattern and d_{hkl} indexation of ZrO2 layer on ZrC particles.
